# Bedside ultrasound measurement of optic nerve sheath diameter in patients with sepsis: a prospective observational study

**DOI:** 10.1186/s13054-020-02959-7

**Published:** 2020-05-18

**Authors:** Ziyue Yang, Cuihong Qin, Shuguang Zhang, Shaohua Liu, Tongwen Sun

**Affiliations:** General Intensive Care Unit, The First Affiliated Hospital of Zhengzhou University, Zhengzhou Key Laboratory of Sepsis, Henan Key Laboratory of Critical Care Medicine, Zhengzhou, 450052 China

## Background

Sepsis-associated encephalopathy (SAE) usually manifests as sleep awakening cycle disturbance, cognitive impairment, delirium, and coma [1]. Considering that brain edema secondary to SAE is one of the complications and causes of death in patients with sepsis, early detection of intracranial hypertension (ICH) is of great significance for timely intervention and improved prognosis. However, most patients with sepsis without intracranial infection have no indications for invasive intracranial pressure (ICP) monitoring; thus, non-invasive ICP monitoring was selected. Bedside ultrasonography measurement of the optic nerve sheath diameter (ONSD) offers a favorable alternative and is presently a new technique [2, 3]. Using this method, we compared the differences in ONSD/ICP between patients with and without SAE, discussed the correlation between ONSD and Glasgow Coma Scale (GCS) score, and evaluated the value of ONSD in predicting the prognosis of patients with sepsis.

## Methods

This study was conducted in the general intensive care unit (GICU) and included patients diagnosed with sepsis from November 2019 to January 2020 [4]. Patients with any of the following criteria were excluded from this study: patients with age < 18, vitreous hemorrhage, eye surgery, central nervous system (CNS) infection, cerebrovascular accident, brain trauma, or previous neurosurgery. If the patient had changes in mental state, especially in consciousness and cognition, and excluding other factors that cause mental changes, the physician in charge decided whether to diagnose the patient with SAE. According to this standard, the patients were divided into three groups: non-SAE group, SAE group, and SAE recovery group, and the ONSD was measured within 24 h of admission. The head of the bed was 30° above the horizontal line, and the ONSD was measured at the retrobulbar 3 mm position, accurate to 0.1 mm.

## Results

**Fig. 1:**
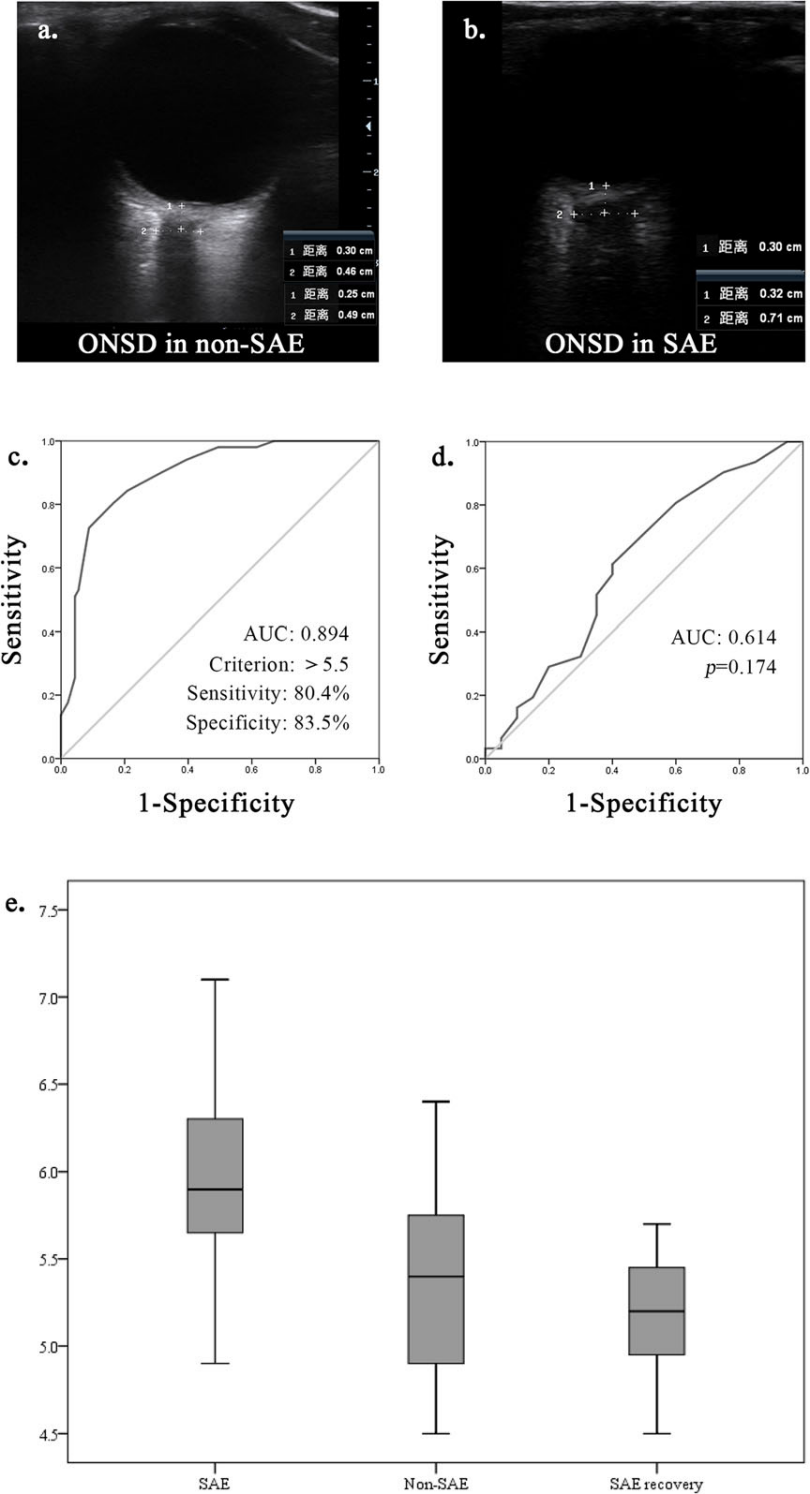
**a** Ultrasonographic images of the optic nerve sheath in a patient with non-sepsis-associated encephalopathy. **b** Ultrasonographic images of the optic nerve sheath in a patient with sepsis-associated encephalopathy (SAE) (ONSD, optic nerve sheath diameter). **c** The receiver operating characteristic (ROC) curve for optic nerve sheath diameter (ONSD) to diagnose septic encephalopathy. **d** The ROC curve for ONSD to predict 28-day survival in patients with sepsis-associated encephalopathy (AUC, area under the curve). **e** Comparison of the optic nerve sheath diameter among the three patient groups (SAE, sepsis-associated encephalopathy)

A total of 142 ONSD ultrasound examinations were performed on 90 patients with sepsis during this trial (non-SAE 71, SAE 51, SAE recovery 20), and the median ONSD in each patient group was 5.1 (4.75–5.4) mm, 5.9 (5.6–6.25) mm, and 5.35 (5.075–5.5) mm, respectively (Table 1, Fig. 1e). The ONSD of patients with SAE was significantly wider than non-SAE patients (Mann-Whitney *U* 395.5, *p* < 0.001) and SAE recovery patients (Fig. 1a, b). After drawing the receiver operating characteristic (ROC) curve (area under the curve, AUC, 0.894, *p* < 0.001), we found the best critical value of ONSD for the detection of SAE in patients with sepsis was ≥ 5.5 mm, with a sensitivity of 80.4% and specificity of 83.5% (Fig. 1c). The ONSD showed a significant negative correlation with the GCS score (rs = − 0.666, *p* < 0.001) and the serum albumin level (rs = − 0.249, *p* = 0.003). Additionally, we found a correlation between ONSD and the bedside angle (0° 5.5 ± 0.5 mm versus 30° 5.1 ± 0.5 mm), even though only 18 patients were observed. In patients with SAE, the ONSD in deceased patients was slightly wider than that in surviving patients (6 [5.7, 6.3] mm versus 5.7 [5.475, 6.2] mm), but there was no statistical difference (*p* = 0.172, Fig. 1d).

**Table 1:** Patient’s characteristics

Characteristic	All sepsis patients^#^	SAE*	Non-SAE*	SAE recovery*
Gender (male, *n*%)	54 (60.0%)	30 (58.8%)	41 (57.7%)	12 (60%)
Age (years, median [IQR])	45 [32, 69]	49 [32, 70]	45 [32, 67]	58 [38, 76]
APACHE II score (median [IQR])	14 [9, 17]	22 [19.5, 28.5]	12 [9, 15]	12.5 [10, 15]
APACHE II score without GCS	14 [9.25, 18.75]	19 [15, 23]	12 [9, 15]	12.5 [10, 15]
SOFA score (median [IQR])	8 [5, 12]	13 [12, 17]	6 [5, 9]	5 [3, 6]
GCS score (median [IQR])	15 [15, 15]	12 [6, 14]	15	15
Serum lactic acid (median [IQR])	2.7 [1.9, 5.1]	6.7 [2.45, 15]	2.1 [1.45, 2.75]	2.4 [2.1, 4.375]
PCT (median [IQR])	3.5 [2.1, 8]	17 [7.5, 36]	2.4 [1.8, 4.5]	2.4 [1.45, 3.3]
Albumin (median [IQR])	30.1 [27.1, 32.1]	28.9 [25.5, 31.05]	30.5 [27.6, 32.2]	34.75 [29.7, 36]
MV time (hours, median [IQR])	33.5 [0, 67]	63 [50, 98.5]	5 [0, 52]	63 [33, 91.5]
In-hospital days (median [IQR])	16 [10, 20]	15 [9.5, 22]	17 [11, 22]	16 [14.25, 24.25]
In-ICU days (median [IQR])	12.5 [9, 18]	12 [9, 20]	15 [9, 20]	14.5 [10.75, 18.5]
28-day mortality (*n*%)	45 (50%)	31 (60.7%)	33 (46.4%)	0
ONSD (mm)	5.4 [5, 5.7]	5.9 [5.6, 6.25]	5.1 [4.75, 5.4]	5.35 [5.075, 5.5]

Data are median [IQR] or *n* (%)

*Non-SAE* sepsis patients with a clear head, without SAE; *SAE recovery* SAE patients who received effective therapy and whose GCS score recovered to 15 points; *APACHE II* Acute Physiology and Chronic Health Evaluation II; *SOFA* Sequential Organ Failure Assessment; *GCS* Glasgow Coma Scale; *PCT* procalcitonin; *IQR* interquartile range; *MV* mechanical ventilation; *ICU* intensive care unit; *ONSD* optic nerve sheath diameter

^#^Patient data collected in the first 24 h after admission

*Patient data collected in the first 24 h after the diagnosis of SAE or SAE recovery

## Discussion

We found that ONSD may be a new diagnostic tool for SAE. There is not enough evidence to show that patients with wider ONSD values have a higher risk of death, and ONSD cannot be used as an indicator for predicting the prognosis of patients with SAE. There are many influencing factors of ONSD; thus, it is necessary to measure it with a unified standard.

## Limitations

ONSD may not be accurate enough, since it is the estimated value of ICP and not the definite value. Moreover, there may be many potential factors affecting the ONSD, which need to be further explored.

## Data Availability

All data generated or analyzed during this study are included in this published article and its supplementary information files.

## Abbreviations

ICP: Increased intracranial pressure
ONSD: Optic nerve sheath diameter
SAE: Sepsis-associated encephalopathy
CNS: Central nervous system
ICH: Intracranial hypertension
GCS: Glasgow Coma Scale
ROC: Receiver operating characteristic
AUC: Area under the curve
